# Supplementary material for: Bub1 positions Mad1 close to KNL1 MELT repeats to promote checkpoint signalling
Source: Nat Commun. 2017 Jun 12;8:15822. doi: 10.1038/ncomms15822 (PMC5472792; doi:10.1038/ncomms15822)
Supplement: Supplementary Information — Supplementary Figures [file ncomms15822-s1.pdf]

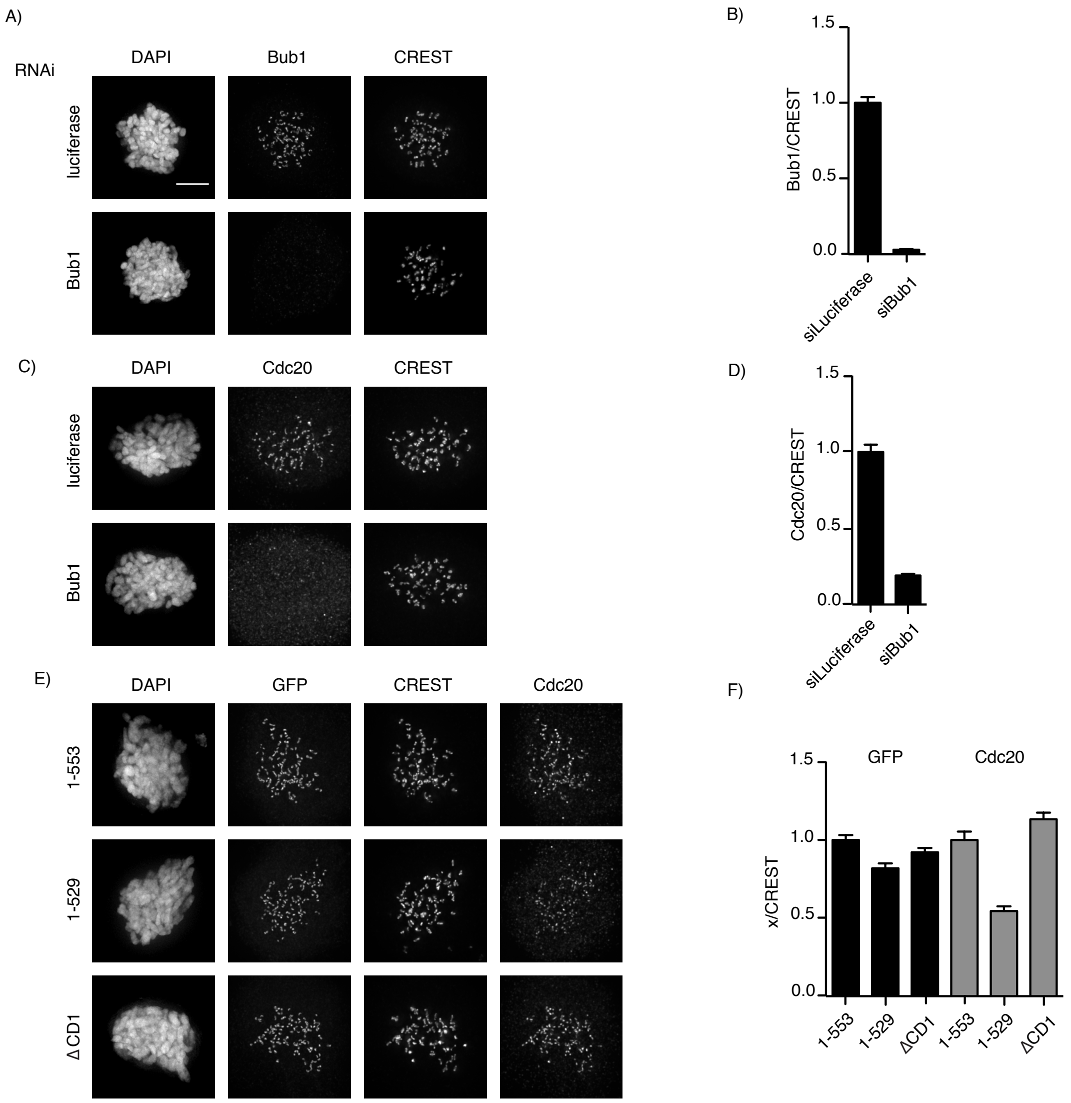


## Supplementary Figure 1

**CD1 domain is not required for Cdc20 kinetochore localization.**

A) HeLa cells were treated with Bub1 RNAi oligos twice within 48 hours before treatment with nocodazole for one hour (200ng/ml). Cells were fixed and stained for Bub1 and CREST. B) Quantification of Bub1 depletion in A). C) Cells treated as in A) except RO3306 (10nM) was applied before the cells were released into medium containing nocodazole for one hour (200ng/ml). Fixed cells were stained for Cdc20 and CREST. D) Quantification of Cdc20 from C). E) HeLa cells were transfected with Bub1 RNAi oligos and RNAi-resistant constructs expressing N-terminal Venus tagged Bub1 1-553, 1-529 or 1-553ΔCD1. Cells were treated similarly as in C) before fixation. Cells were stained for GFP, CREST and Cdc20. F) Quantification of GFP and Cdc20 intensity on kinetochores from E). At least 200 individual kinetochores from ten cells were measured in each condition. The mean with standard error of mean is indicated. Scale bar, 5 µm.

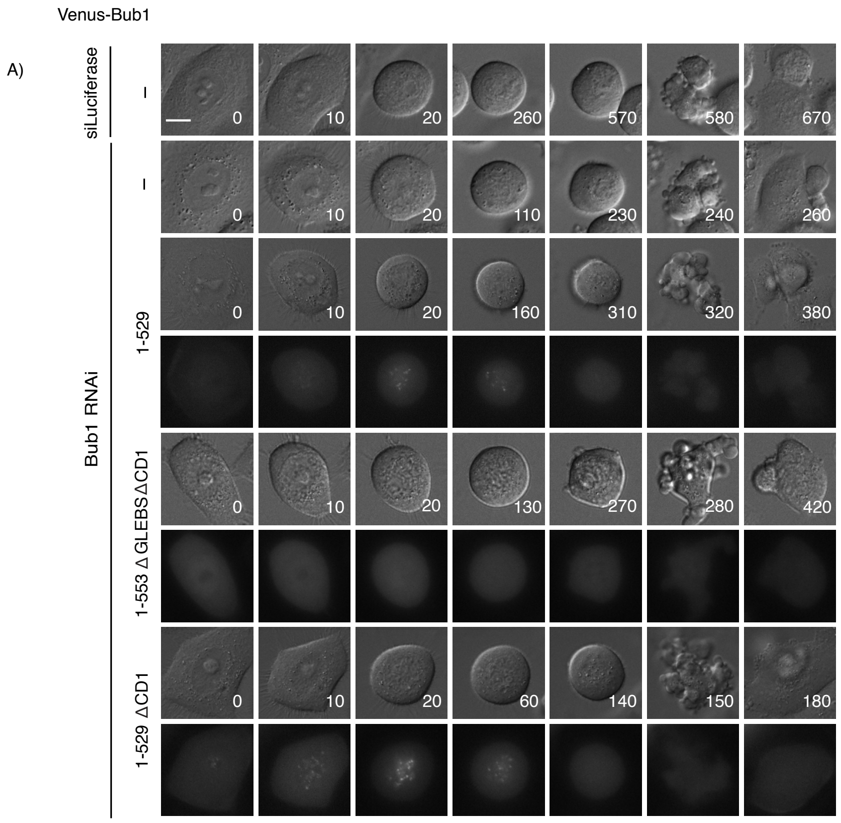


**Supplementary Figure 2**

**CD1 domain is important for SAC activity in HeLa cells.**
A) Representative still images of live cell imaging for Fig. 1C. Scale bar, 5 µm.

A)

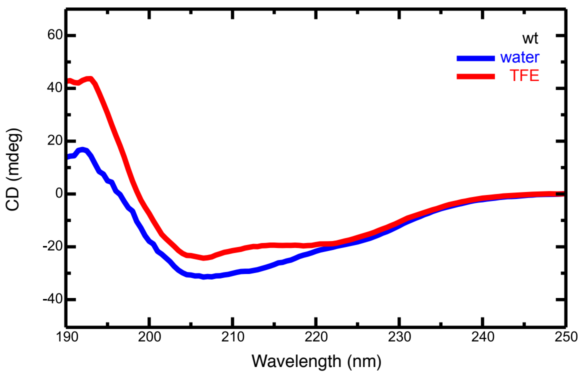


B)

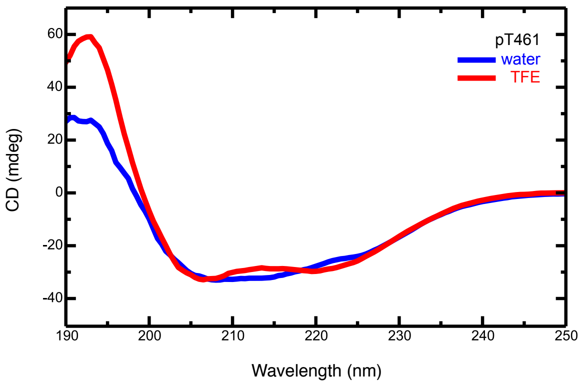


## Supplementary Figure 3

**Circular dichroism spectra of CD1 peptides**
CD spectra of a Bub1 CD1 wild-type peptide or a CD1 peptide phosphorylated on T461. Peptide sequences are indicated in Fig 4.

A)

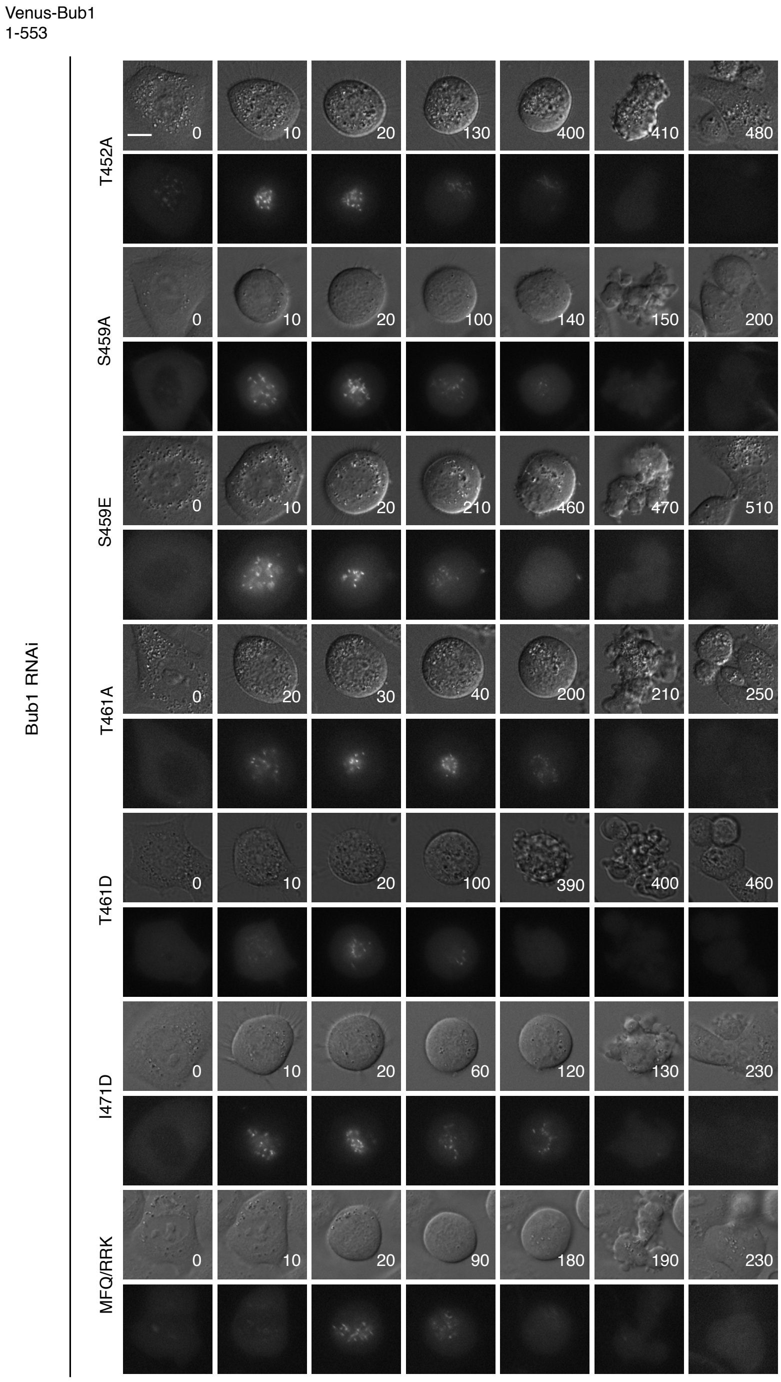


B)

| | Control | Cdk1-cyclin B1 | Mps1 |
|---|---|---|---|
| 456-VQPS(Phos)PTVHTK | 0 | 5,59E+08 | 0 |
| 456-VQPSPT(Phos)VHTK | 0 | 1,89E+08 | 7,84 E+08 |

**Supplementary Figure 4**

**Mutational analysis of CD1**

A) Still images of live cell imaging for Fig. 1G. Scale bar, 5 µm.

B) The indicated phospho-peptides detected by mass-spectrometry of GST-Bub1 425-500 phosphorylated with the indicated kinases. The localization probability of the phosphorylation sites was > 0,97 and the peptide intensities are indicated for each condition.

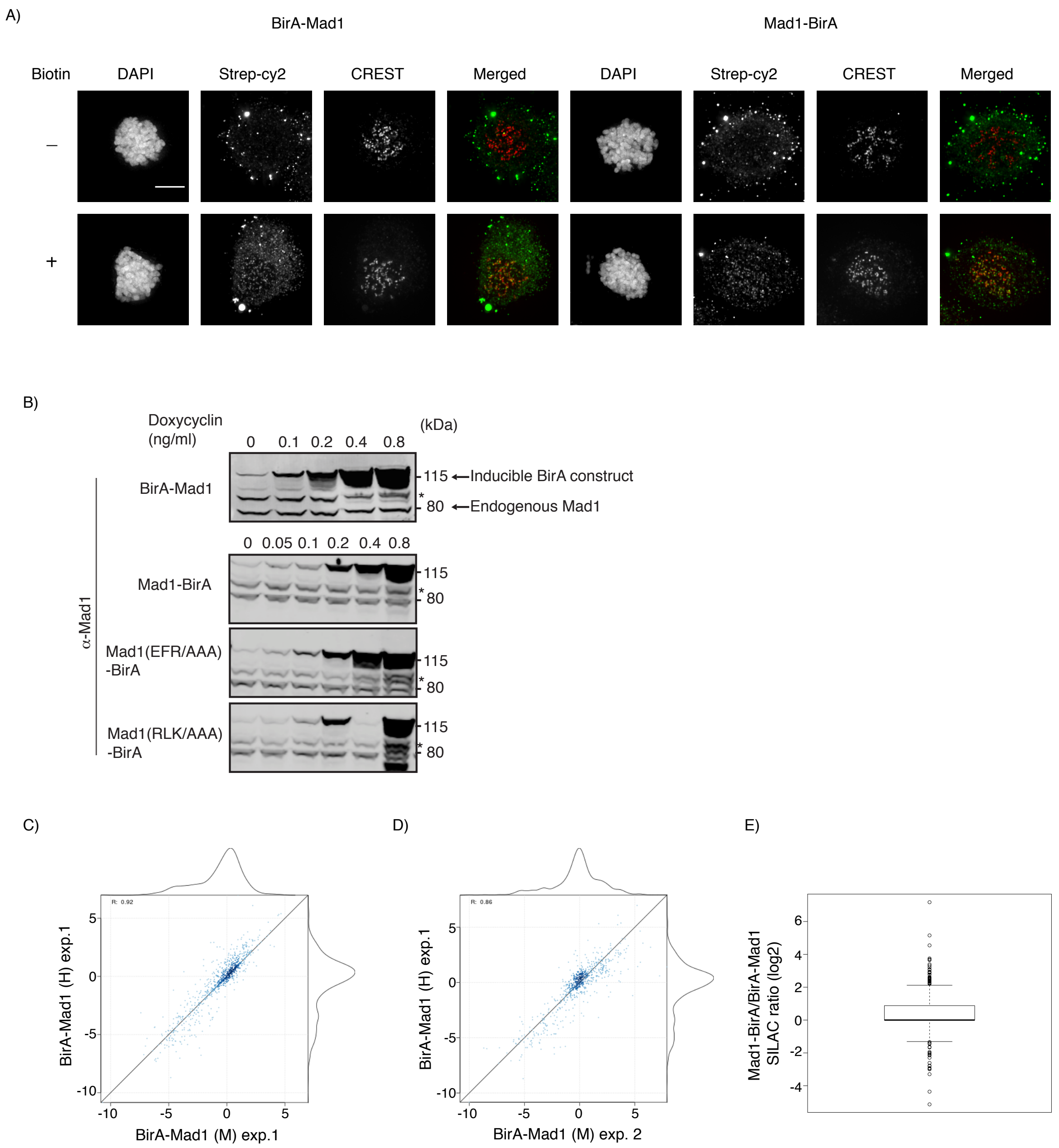


## Supplementary Figure 5

**BirA-Mad1 and Mad1-BirA fusion proteins biotinylate kinetochores in mitotic cells.**
A) Stable cell lines expressing BirA-Mad1 or Mad1-BirA were induced by doxycyclin for 48 hours. Biotin (25 µM) was added to cells treated with nocodazole for 6 hours where indicated. After fixation, cells were stained by DAPI, FITC conjugated streptavidin and CREST. Scale bar, 5 µm. B) Nocodazole arrested HeLa cells stably expressing either Mad1-BirA or BirA-Mad1 fusion proteins were exposed to a series of doxycyclin concentrations as indicated for 18 hours to obtain near endogenous expression levels of the BirA fusion constructs. * cross reacting band. C-D) Correlation of replicate BirA-Mad1 experiments demonstrates good correlation between experiments with Pearson coefficients of 0.92 and 0.86 respectively. E) Boxplot analysis of H/M (Mad1-BirA/BirA-Mad1) SILAC ratios

A)

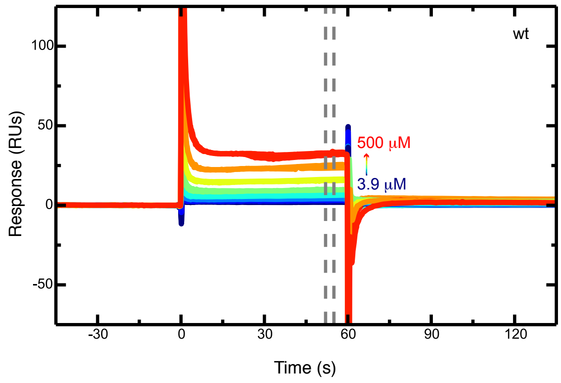


B)

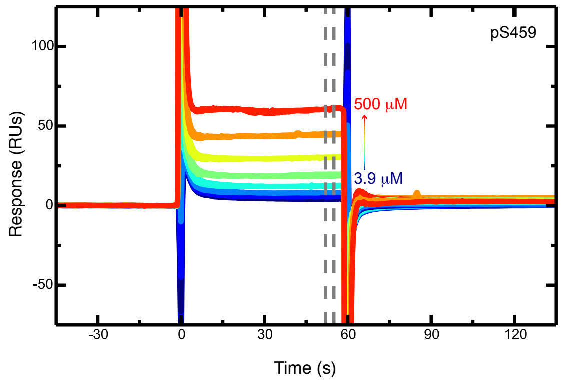


C)

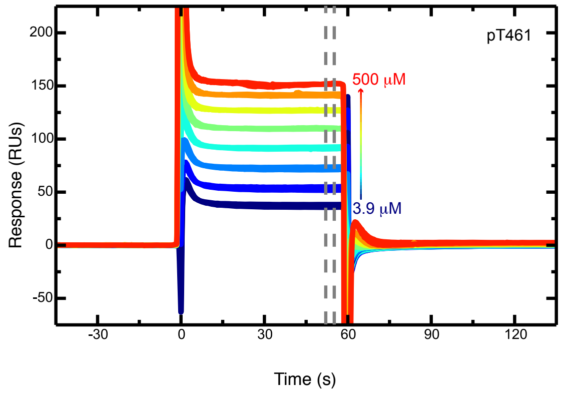


**Supplementary Figure 6**

**Surface plasmon resonance raw data**

A-C) Sensorgrams for Mad1 binding to CD1 peptides (A: WT, B: pS459, C:pT461). Mad1 was injected at increasing concentrations starting at 3.9 µM up to a highest concentration of 500 µM. Steady state responses (averaged between discontinuous grey lines) were used to determine the equilibrium dissociation constants by fitting the data to a steady state affinity model.

A)

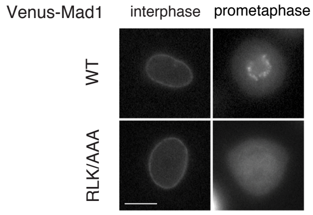


B)

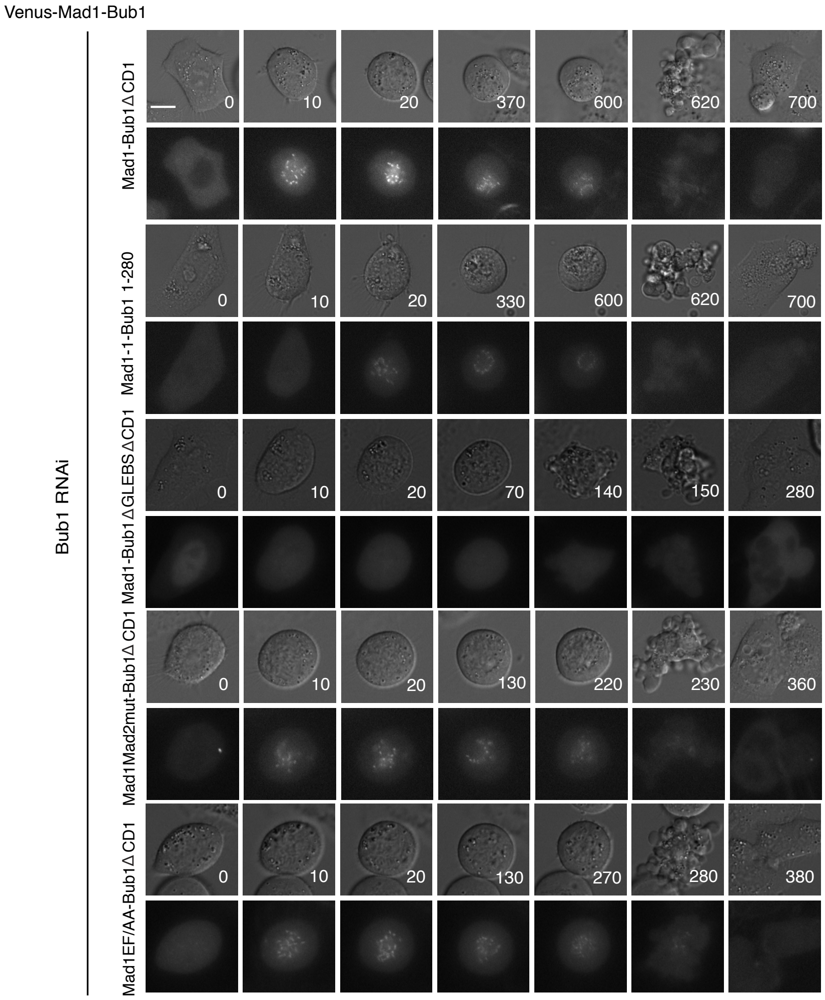


## Supplementary Figure 7

### A Mad1-Bub1 fusion protein bypasses the requirement for CD1

A) Localization of Venus Mad1 and Venus Mad1 RLK/AAA in HeLa cells depleted of endogenous Mad1 analyzed by live cell imaging. Scale bar, 5 µm

B) Still images for Fig. 5A and Fig. 6C. Scale bar, 5 µm.

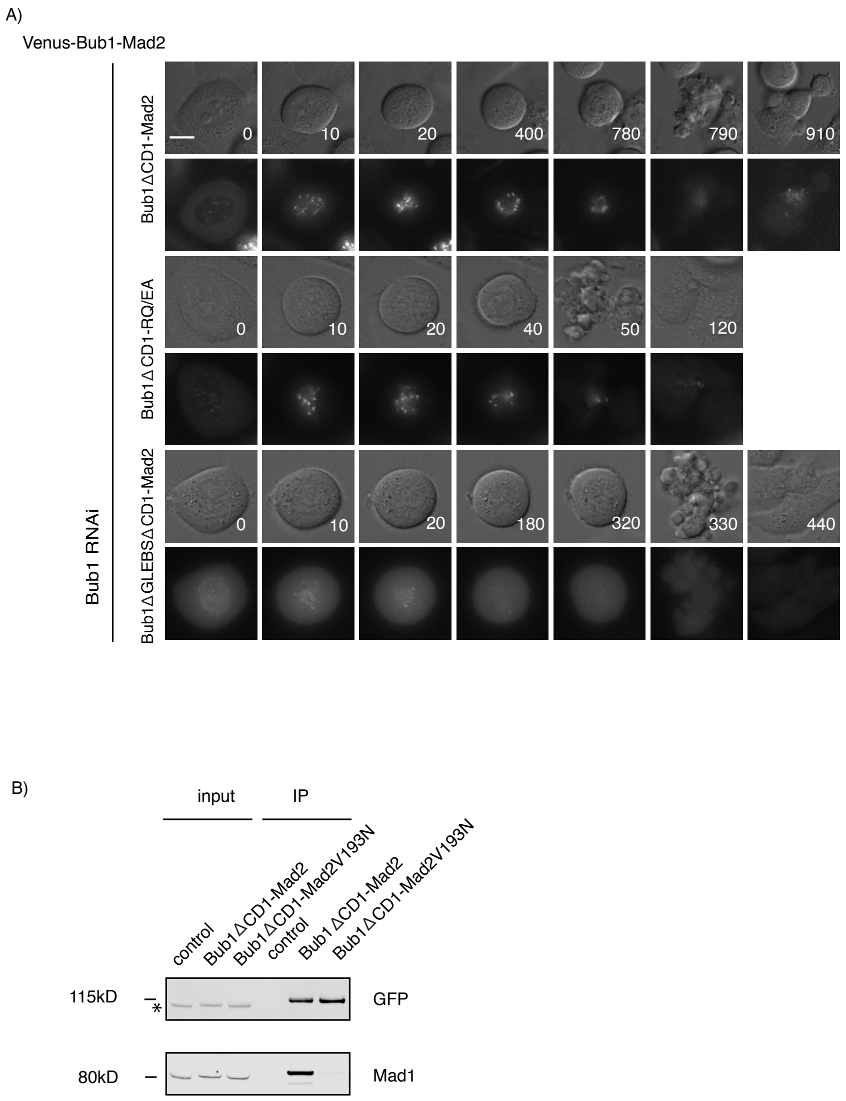


## Supplementary Figure 8

**A Bub1-Mad2 fusion protein bypasses the requirement for CD1.**
A) Still images for Fig. 5B. Scale bar, 5 µm. B) HeLa cells were transfected with Bub1 ΔCD1-Mad2 contructs for 48 hours before nocodazole treatment (200ng/ml). Mitotic cells were collected and immunoprecipitation was performed using GFP trap and the level of associated Mad1 was determined by quantitative western-blot. (Representative of two independent experiments). * Unspecific band in input recognized by GFP antibody.

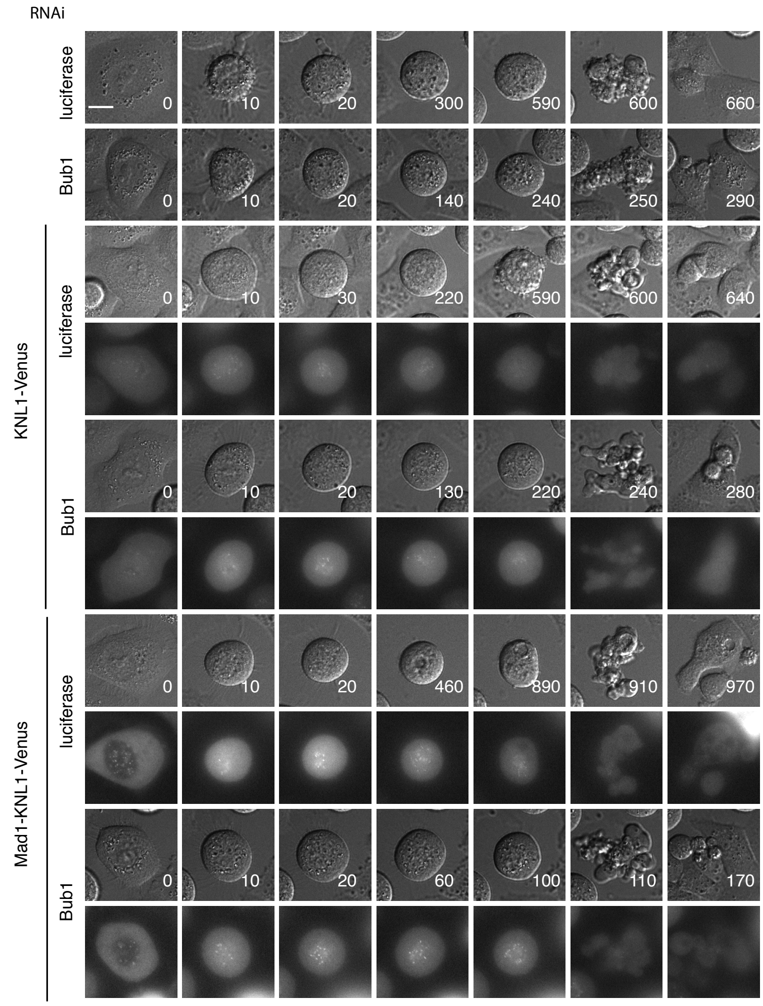


**Supplementary Figure 9**

**Bub1 is still required for SAC activity when Mad1 is tethered to KNL1**
Still images of live cell imaging of Fig. 5C. Scale bar, 5 µm.

A)

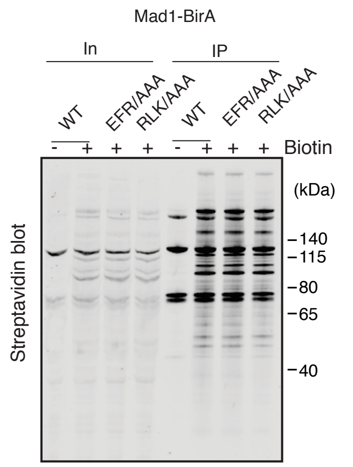


**Supplementary Figure 10**

**Biotinylated proteins from wild type Mad1-BirA or mutant Mad1-BirA.**
Input for purifications in Fig. 6B .

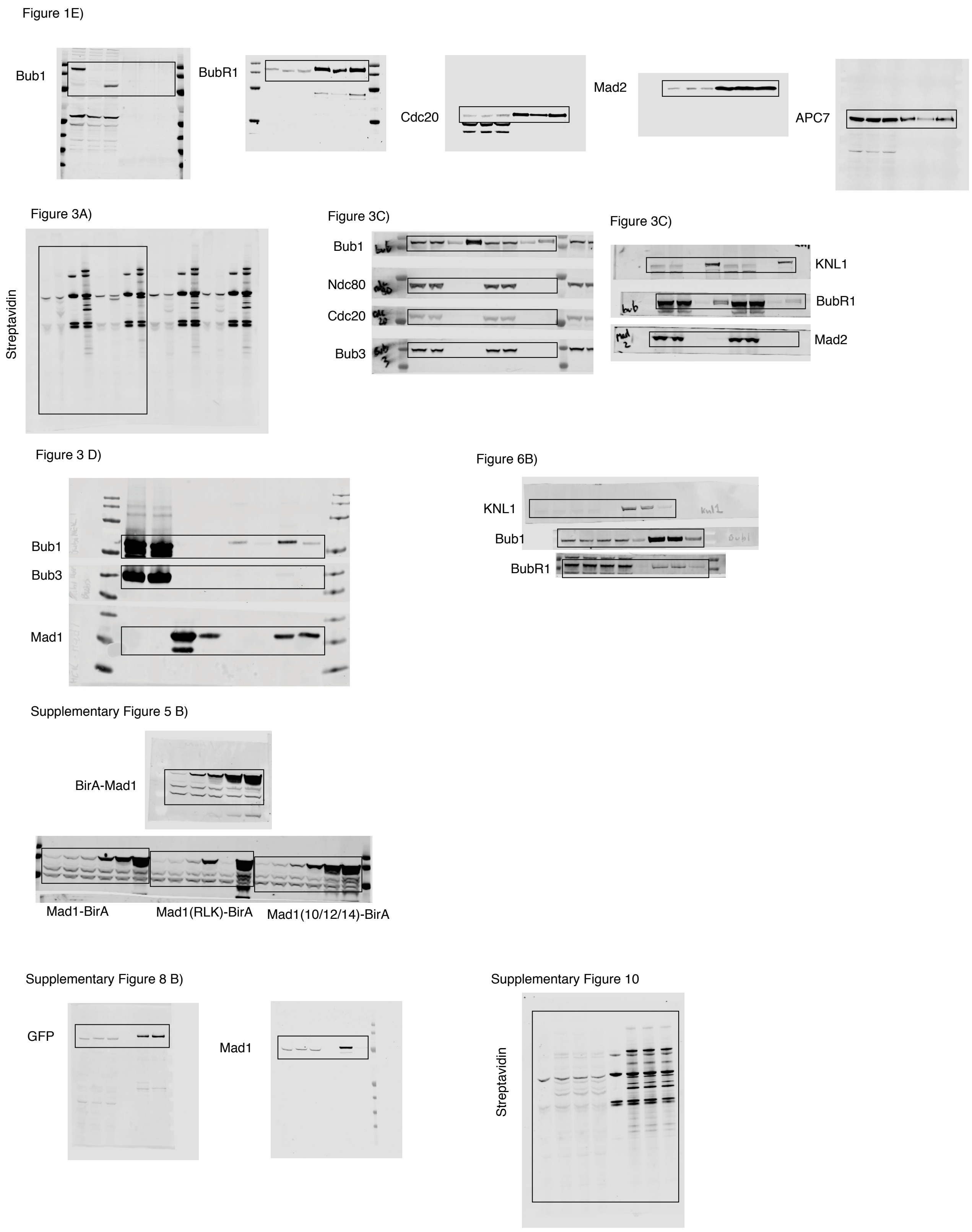


## Supplementary Figure 11

## Uncropped images of western blots

Uncropped images of western blot presented in above figures. Black boxes show the region cropped from each blot and presented in above figures.
